# Predictive Model of Clinical Attachment Loss and Oral Health-Related Quality of Life through Depressive Symptomatology, Oral Hygiene Habits, and Proinflammatory Biomarkers: A Pilot Study

**DOI:** 10.3390/dj8010020

**Published:** 2020-02-21

**Authors:** Norma Idalia Rodríguez Franco, José Moral de la Rubia, Andrea Guadalupe Alcázar Pizaña

**Affiliations:** 1 Facultad de Odontología, Universidad Autónoma de Nuevo León, Monterrey N.L. 66460, Mexico; 2 Facultad de Psicología, Universidad Autónoma de Nuevo León, Monterrey N.L. 66460, Mexico; jose.morald@uanl.edu.mx; 3 Centro de Investigación y Desarrollo en Ciencias de la Salud, Universidad Autónoma de Nuevo León, Monterrey N.L. 66460, Mexico; andrea.alcazarpzn@uanl.edu.mx

**Keywords:** periodontitis, quality of life, depressive disorder, oral hygiene, biomarkers, interleukin-1β, interleukin-6, matrix metalloproteinase-8

## Abstract

Subjective aspects such as oral health-related quality of life (OHRQoL) and depression are important aspects in the periodontal care. The objectives of the study were to test a predictive model of clinical attachment loss and OHRQoL in a pooled sample of dental patients with periodontitis and mental health patients with depressive symptomatology, and test the invariance of the model across both types of patients. Three self-report scales were applied to assess depression, OHRQoL and oral hygiene habits, saliva samples were collected for three proinflammatory biomarkers, and the clinical attachment loss was measured in 35 patients with periodontitis and 26 patients with depressive symptomatology. Data were analyzed through structural equation modeling. The one-group analysis revealed a psychosomatic complaint model of disagreement between the complaint and the clinically observable. In the multi-group analysis, the model was not invariant. It was necessary to introduce a singularity in relation to depressive symptomatology for each population. Thus, a good and equivalent fit was achieved between the six nested models in constraints, as well as equivalent parameters between both types of patients. The study of a dental population in conjunction with a mental health population with a psychosomatic risk factor reveals interesting and unexpected results.

## 1. Introduction

### 1.1. Mind and Body Interaction

The study of the interactions between mind, body and health have demonstrated the existence of reciprocal relationships between the central, autonomic, and neuroendocrine nervous systems. On the one hand, emotional states can affect immunity. For example, depressive symptomatology can induce an increase in proinflammatory regulators, such as interleukin 1 beta (IL-1β) and interleukin 6 (IL-6). In turn, elevations of proinflammatory cytokines (IL-1β and IL-6) can alter the activity of the central nervous system, contributing to the appearance of depressive symptoms [1].

Depression → increase in proinflammatory cytokines → decrease in immunological competence → occurrence or aggravation of infectious or tumor diseases is one of the most studied sequential paths of the effect of the mind on health [2]. Among the proinflammatory markers, there is clear evidence that interleukin 6 (IL-6) and C-reactive protein regulate the hypothalamic-pituitary-cortico-adrenal system and its secretion is stimulated by physical or psychological stressors [3]. These two immunological markers are increased in people with depression compared to people without depression [4] and are reduced after two weeks of treatment with antidepressant drugs [5]. Another cytokine that increases in the depressive state and affects immunological competence is IL-1β [1,6].

### 1.2. Depression and Periodontal Disease

Chronic periodontitis is defined as an infectious disease that results in inflammation of the supporting tissues of the tooth, loss of progressive insertion, bone loss, formation of periodontal pockets, and gingival recession [7].

In a study with participants from Brazil, Australia and Denmark, it is concluded that depressive symptomatology is a risk factor with a small effect size on periodontal disease [8]. In another study conducted in Japan, it is pointed out that depressive disorders are related to a decrease in oral health care consultations and an increased risk of periodontal disease and tooth loss; In addition, early onset mental disorders increase the probability of suffering from oral diseases over time [9]. However, in a systematic review of the literature and meta-analysis on the association between depression and periodontitis, great heterogeneity was found between the studies and the size of the effect, so it could not be concluded that the association was significant [10].

In periodontal disease, depression has been found to reduce the immune response favoring infection by bacteria with a high degree of destruction of periodontal tissue [11]. On the other hand, it has been found that the salivary levels of IL-1β, IL-6, and matrix metalloproteinase-8 (MMP-8) are significantly elevated in patients with periodontitis compared to healthy people, so it has been proposed that the elevation of these three salivary biomarkers associated with inflammatory and destructive processes may constitute indicators of the severity of periodontitis [12]. Consequently, the elevation of proinflammatory markers induced by depression may be a route that affects the course of periodontal disease due to a decrease in immunological competence [11]. Another route of the effect of depression on periodontitis is oral hygiene. Poor oral hygiene as a result of depressive apathy may favor plaque accumulation and, over time, the appearance of gingivitis and periodontal disease [13].

### 1.3. Depression and Oral Health-Related Quality of Life

The loss of periodontal support structure has negative effects on masticatory function and OHRQoL; Understanding by OHRQoL the comfort or convenience that people experience when they eat, sleep and get involved in social interaction, as well as positive assessment and satisfaction with oral health [14]. In turn, it has been observed that the severity of depression has a negative impact on OHRQoL [15], which can be attributed to a direct effect due to cognitive bias towards the negativity associated with depression [16], as well as an indirect effect mediated by a deterioration of periodontal health through decreased immunological competence and poor oral hygiene habits [11,17].

### 1.4. Study Approach

The objectives of the present study were two. The first objective was to contrast a model to predict clinical attachment loss and OHRQoL. In this model, clinical attachment loss is predicted by the direct effect of oral hygiene habits and depressive symptomatology; and OHRQoL is predicted by direct effect of clinical attachment loss, depressive symptomatology, and increased proinflammatory immune response. The second objective was to contrast the invariance of the model between the two types of patients that integrated the sample: dental patients with periodontitis and mental health patients with depressive symptomatology. The former were chosen because their clinical condition is defined by the clinical attachment loss [7], and the latter because their clinical condition is defined by one of the most important psychosomatic risk factors such as depression [11]. The hypotheses stated to specify the model were the following five:

1. Proinflammatory immune response as a latent variable measured by the biomarkers IL-1β, IL-6, and MMP-8 [1,3,6] has convergent validity; this type of validity is understood as the degree of certainty that the proposed indicators measure the same variable [18].
2. A greater depressive symptomatology is a predictor of poor oral hygiene habits [13], clinical attachment loss [8,9,11], loss of OHRQoL, and increased proinflammatory immune response [1,2].
3. Poor oral hygiene habits are predictors of clinical attachment loss [19].
4. Clinical attachment loss is a predictor of OHRQoL deterioration [14] and an increase in proinflammatory immune response [12].
5. The increase in proinflammatory immune response is a predictor of OHRQoL deterioration [20].

## 2. Materials and Methods

### 2.1. Sample and Sample Procedure

A study was conducted with a non-experimental cross-sectional design. An incidental non-probabilistic sampling was used in two patient populations: dental patients with periodontitis and mental health patients with depressive symptomatology. The inclusion criteria were: reside in Monterrey or its metropolitan area (Nuevo León, Mexico); as well as being between 35 and 65 years old due to the prevalence of chronic periodontitis [21], but excluding adults 65 years of age or older due to their more complex oral health problems [22]. For the sample of dental patients, it was added to receive the diagnosis of periodontitis and not to have initiated any periodontal treatment, including anti-inflammatory drugs. For the sample of mental health patients, it was added to suffer from a major depressive disorder (diagnosed by a psychiatrist) or to present depressive symptomatology (total score in the Beck Depression Inventory-II ≥ 14 in patients treated in a psychology clinic). Not being under antidepressant medication or having less than two weeks of having initiated it (window period for the clinical effect of antidepressants) [5]. The exclusion criteria were: pregnant woman due to hormonal changes [23], suffering from diabetes or some other systemic disease because it is a risk factor that contributes to the severity of periodontitis [24], using orthodontic appliances because it favors dental plaque accumulation [25], and being a smoker because it constitutes a risk factor for periodontitis [26]. The elimination criterion was not to attend the periodontal evaluation appointment, which was scheduled after the application of the questionnaire.

The sample of dental patients was made up of 35 patients diagnosed in the Department of Periodontics of the Faculty of Dentistry of the Universidad Autónoma de Nuevo León (UANL). The mental health sample was made up of 10 patients diagnosed with major depressive disorder; six of them from the Department of Psychiatry of the University Hospital of the UANL and four from the Psychiatric Rehabilitation Unit of the Secretary of Health of the State of Nuevo León. The patients diagnosed with depressive symptomatology were 16 and came from the Psychological Services Unit of the Faculty of Psychology of the UANL. The psychiatric patients first went to their psychiatric office. When they were diagnosed with major depression, we invited them to participate in the study the same day of their consultation, whereas they were not taking antidepressant medications. In case they were already taking antidepressant medications, their participation in the study should be within the first two weeks of the start of treatment. No patient in the psychology clinic received antidepressant drugs.

The sample size was calculated before starting the study. For this purpose the Priori Sample Size Calculator for Structural Equation Models created by Preacher and Coffman [27] was used. Testing the null hypothesis of root mean square error of approximation (RMSEA) = 0 (value of a good fit to the data) against an alternative hypothesis of RMSEA = 0.10 (value of a poor fit) with a level of significance of 0.05 and 10 degrees of freedom (hypothesized model) requires at least 113 participants to reach a power of 0.60. The thumb rule of at least five participants per estimated parameter [28] suggest a minimum sample size of 90 (90:18 = 5:1). It was not possible to reach this minimum due to the exclusion criteria in the sample of mental health patients, especially in relation to the use of antidepressants. We decided to use repetitive sampling (bootstrap) procedures, which are the alternative recommended in this situation [29]. It should be noted that the proportion of five participants per estimated parameter is reached in the pooled sample (58:12 ≈ 5:1). The present investigation suffers from low potency despite its efforts in data analysis to overcome the limited sample size, and consequently, should be considered as a pilot study.

### 2.2. Measuring Instruments

The clinical attachment loss [7,21] was measured as the periodontal parameter to be included in the model. A 15 mm periodontal probe was used. A single examiner performed the evaluation. The distance in millimeters between the amelocementary line and the base of the periodontal pocket was measured. It was full mouth examination. In each tooth, six sites (three buccal and three palatine or lingual) were evaluated. The value reported per participant corresponds to the average of sites with attachment loss (≥1 mm). The patients had most of their dental pieces in the fourth quadrant, but the number of missing teeth was not recorded.

Probing depth and gingival bleeding index were also assessed. These two periodontal parameters were used for descriptive purposes in this study and were not included in the model. One option could have been to specify a latent variable named ‘periodontitis’ with three indicators (bleeding on probing, probing depth, and clinical attachment loss). However, this would mean a significant loss of power when it is already low, and thus it decided to focus the model only on the clinical attachment loss, that is, on the severity of periodontitis. Probing depth was measured through the distance in millimeters between the gingival margin and the bottom of the periodontal pocket. In each dental piece, six sites (three vestibular and three palatine or lingual) were evaluated. The value reported per participant corresponds to the average probing depth (≥4 mm depth). The probing depth was classified into three ordered categories: 4 mm shallow, 5 mm moderate, and ≥6 mm deep [21]. It was observed if bleeding occurs at 10 s after a soft probing at the entrance of the gingival groove. If there was bleeding, it was recorded as a positive site. The gingival bleeding index was obtained by dividing the number of positive sites by the number of sites examined and multiplying the result by 100 [30].

Saliva samples were collected in scheduled appointments at morning. Total unstimulated saliva was collected. Patients were asked to refrain from eating, drinking, or performing oral hygiene procedures for at least 1 h before collection. Patients rinsed their mouth with water, and expectorated saliva into a sterile microtube, using a glass funnel [31]. Saliva samples were frozen a −80 °C. The day the samples were analyzed, brought to room temperature, vortexed, and centrifuged at 1500× *g* for 15 min.

The three proinflammatory biomarkers were measured in saliva samples, applying the enzyme-linked immunoabsorption assay (ELISA). The ELISA Kit. SALIMETRICS^®^ (sandwich type, Carlsbad, CA, USA) was used to quantify IL-1β and IL-6, and the Human MMP8 SimpleStep ELISA^®^ Kit (Abcam219050, Cambridge, UK) to quantify MMP-8. Total expectorated saliva was collected. The samples were frozen at −80 °C. A filter at 450 nm was used in the plate reader that yielded the measurements in pg/mL after 10 min of exposure once the sulfuric acid solution was added. The kits reach a sensitivity of 0.37 pg/mL for IL-1β [32], 0.07 for IL-6 [32], and 19.5 for MMP-8 [33]. The analysis to estimate the concentration of proinflammatory biomarkers in saliva samples was performed at the Center for Research and Development in Health Sciences, UANL.

A questionnaire consisting of informed consent, questions about sociodemographic information and three self-report scales was applied:

Beck Depression Inventory-II (BDI-II) [26]. It consists of 21 items that are answered by choosing four closed response options that are scored from 0 to 3. Four levels of depressive symptomatology are distinguished based on the BDI-II total score: between 0 and 13 minimum, between 14 and 19 mild, between 20 and 28 moderate, and between 29 and 63 severe [34]. The BDI-II has been validated in Mexico. It has shown excellent internal consistency reliability in a sample of 420 medical students (α = 0.92) and good in a community sample of 220 adults from Mexico City (α = 0.87); a three-factor structure (negative attitude, performance difficulties, and somatic elements) showed the best fit to the data [35].

The Oral Hygiene Habits Scale (OHHS) [36] consists of eight direct items with five ordered response categories that are scored from 0 to 4. By adding the scores obtained in the items and dividing by 8, the OHHS total score is obtained in a continuum from 0 to 4. Higher score reflects better oral hygiene habits. There are three levels of oral hygiene habits based on the OHHS total score: between 0 and 0.99, bad; between 1 and 2124, regular; and between 2125 and 4, good. In Mexico, its internal consistency reliability was good (ordinal α = 0.83 among 256 adults in the general population and 0.87 among 240 dental patients) and it had two-factor structure: dental brushing and flossing [36].

The Oral Health Impact Profile applied to Periodontal Disease (OHIP-14-PD) [37] consists of 14 direct items with five ordered categories of response (from 0 = never to 4 = very frequently). This instrument evaluates functional limitation, physical pain, psychological discomfort, physical disability, psychological disability, social disability, and disability. The total score is obtained by the direct sum of the 14 items and has a range from 0 to 56. The OHIP-14-PD total score is classified as: 0–9, low impact; 10–23, medium; and 24–56, high. The OHIP-14-PD showed a one-dimensional structure with adequate invariance between 256 general population persons and 249 dental patients, as well as an excellent internal consistency reliability (ordinal α = 0.93) in the pooled sample of 505 Mexican participants [37].

### 2.3. Ethical Aspects

Informed written consent was requested and the information was kept strictly confidential in accordance with the Regulations of the General Health Law on Research Subject for Health [38]. The research was approved by the Research Ethics Committee of the Psychiatric Rehabilitation Unit (registration key CONBIOÉTICA 19CEI01720130828) on 1 August 2016. The research was registered in the Department of Education, Health Research and Quality of the Health Services of Nuevo León with registration number DEISC-19 01 16 16. People who were detected periodontitis were offered treatment in the Periodontics Department of the Faculty of Dentistry of the Universidad Autónoma de Nuevo León.

### 2.4. Analysis of Data

Statistical analyzes were done with the Statistical Package for Social Sciences (SPSS) version 24 (Chicago, IL, USA), program for the Analysis of Moment Structures (AMOS) version 16 (Chicago, IL, USA), and Microsoft Excel 2013 (Bell Gardens, CA, USA). The two-tailed tests with a significance level of 0.05 were performed. Due to the incidental nature of the samples, the randomness of the data sequence (in its order of collection) was tested through the Wald–Wolfowitz runs test for randomness; this was done to justify the use of inferential statistics. The reliability of the cytokine estimates was calculated by the correlation between eight values of the antibody’s serial concentration and the average absorbancy (negative of the base ten logarithm of the quotient between the intensity of the transmitted light and the intensity of the incident light in the sample under the microscope). For cytokines, a Pearson product-moment correlation coefficient that include the one in its interval estimate with a 95% confidence level was required. Since the correlations were calculated between eight pairs of data, their confidence interval was estimated through percentile bootstrapping method with the simulation of 1000 random samples. The internal consistency reliability of scores in the psychometric scales was estimated using ordinal coefficient alpha. An ordinal α value between 0.70 and 0.79 reflects acceptable reliability, between 0.80 and 0.89 good, and ≥0.90 excellent.

At the univariate level, the adjustment of the scores to a normal distribution was tested by the Kolmogorov–Smirnov test with the Lilliefors correction in the pooled sample and by the Shapiro–Wilk test in the two patient samples. In addition, it was tested by the D’Agostino–Pearson test in all of them. The assumption of normality at the multivariate level was verified by a value in the Mardia’s standardized coefficient of multivariate kurtosis < 3 [39]. Mathematical transformations were used through the Napierian logarithm to achieve a good adjustment to normality in cytokine distributions.

The hypothetical model was tested by structural equation modeling. The parameter estimation was done by maximum likelihood by having a good approximation to multivariate normality. Goodness of fit was assessed using seven indices: likelihood ratio chi-square test (χ^2^), Bollen–Stine bootstrap probability value with the simulation of 1000 random samples (BS *p*), relative or normed chi-square (χ^2^/df), goodness-of-fit index (GFI), incremental fit index (IFI) through Bollen’s coefficient delta-2, comparative fit index (CFI), root mean square of error of approximation (RMSEA), and the standardized root mean of square residual (SRMR). P(χ^2^ > _1−α_χ^2^_df_) and BS *p* > 0.05, χ^2^/df ≤ 2, GFI, IFI, and CFI ≥ 0.95, AGFI ≥ 0.90, as well as RMSEA and SRMR ≤ 0.05 show a good fit. P(χ^2^ > _1−α_χ^2^_df_) and BS *p* > 0.01, χ^2^/df ≤ 3, GFI, IFI and CFI ≥ 0.90, AGFI ≥ 0.85, as well as RMSEA ≤ 0.08 and SRMR < 0.10 reflect an acceptable fit [39].

To establish the convergent validity of the measurement model, three criteria were considered: measurement weights, average variance extracted (AVE), and composite reliability coefficient (ω). Measurement weights ≥ 0.50, ω ≥ 0.70 and AVE ≥ 0.44 indicate an acceptable convergent validity level with three indicators [18].

The invariance of the model (reduced to its significant paths) was tested across the two types of patients through multi-group analysis, specifying six nested models in constraints: Unconstrained model (UC), with constraints on measurement weights (MW), on structural weights (SW), on structural covariances (SC), on structural residuals (SR), and on measurement residuals (MR). The equivalence of parameters between both patient samples in the six nested models was tested by the Z-test and the equivalence of goodness of fit by the chi-square difference test [39].

## 3. Results

### 3.1. Sample Description

The frequency of participants was statistically equivalent between both patient samples by the binomial test (*p* = 0.306, exact two-sided *p*-value). At the same time, the frequencies of both sexes were statistically equivalent between both samples by the chi-square test with Yates correction (χ^2^[1, *N* = 61] = 0.44, *p* = 0.505, asymptotic two-sided *p*-value). The proportion of both sexes it was statistically equivalent in the pooled sample by the binomial test (*p* = 1, exact two-sided *p*-value), therefore half of the participants are women and the other half men. The frequencies of the five categories of civil status were statistically equivalent between both samples by the chi-square test (χ^2^[4, *N* = 61] = 6.44, *p* = 0.177, exact two-sided *p*-value). Levels of schooling and subjective socioeconomic status were statistically equivalent using the Mann–Whitney U-test (Z_U_ = −0.05, *p* = 0.958, and Z_U_ = −1.31, *p* = 0.191, respectively) and the average age using Student’s *t*-test (t [59] = 1.43, *p* = 0.159). The average age in the pooled sample was 46.36 years (SD = 7.57) with a minimum of 34 and a maximum of 63. However, there were differences in occupation (χ^2^[2, *N* = 61] = 6.98, *p* = 0.030 exact bilateral). When performing the pairwise comparisons applying the Bonferroni correction, there was only a significant difference in the category of blue-collar employees. The frequency of unskilled workers/technical workers was higher in the sample of dental patients than in that of mental health patients (Table 1).

In the sample of dental patients, 51.4% (18 out of 35) of participants had severe periodontitis (5 mm or more of clinical attachment loss) and 48.6% (17 out of 35) moderate (3–4 mm). Regarding extension of periodontitis, 97.1% had generalized periodontitis (≥30% of the sites are involved with clinical attachment loss ≥ 1 mm) and 2.9% localized (<30%). In the sample of mental health patients, 7.7% (2 out of 26) of participants had severe periodontitis and 92.3% (24 out of 26) had moderate periodontitis. There was not absence or mild cases of periodontitis in either of the two samples. Regarding extension of periodontitis in mental health patients, 50% had generalized periodontitis and 50% localized. The severity (χ^2^[1, *N* = 58] = 9.28 with Yates’s correction for continuity, *p* = 0.002 two-tailed asymptotic probability) and extension of periodontitis (χ^2^[1, *N* = 58] = 17.46 with Yates’s correction for continuity, *p* < 0.001) were significantly higher in dental patients than in mental health patients.

The average gingival bleeding index was significantly higher (Welch’s t [46.37] = 3.78, *p* < 0.000) in dental patients (*M* = 15.41%, 95% CI: 10.13, 20.69) than in mental health patients (*M* = 4.67%, 95% CI: 2.29, 7.06). Moreover, the average probing depth was significantly greater (Welch’s t [46.37] = 3.78, *p* < 0.001) in dental patients (*M* = 4.57, 95% CI: 4.47, 4.66) than mental health patients (*M* = 4.27, 95% CI: 4.19, 4.35).

### 3.2. Randomness of the Analyzed Variables

When using the mean as a cut-off point, the assumption of randomness was maintained in the two patient samples with a significance level of 0.05 for all variables. In the pooled sample, the assumption was also maintained with this level of significance, except for the variables IL-1β and clinical attachment loss that should be reduced to 0.01. Although if the median with clinical attachment loss (*Z* = 1.42, *p* = 0.156) and the mode with IL-1β (*Z* = 0.19, *p* = 0.850) are used as the cut-off point, it would remain with a significance level of 0.05 (Table 2).

### 3.3. Reliability of Estimates of IL-1β, IL-6, MMP-8, and Scores in Psychometric Scales

The correlations between the average absorbancy and the antibody concentrations for IL-1β, IL-6 and MMP-8 in the pooled sample varied from 0.97 to 1. In their 95% confidence interval estimation, the three correlations included 1, that is, they were unitary. For these measurements, both samples were joined (58 patients for IL-1β, and 59 for IL-6 and MMP-8), since each kit had a 96-well microplate. Three cases for IL-1β and two for MMP-8 were eliminated by invalid estimates.

In the pooled sample of 61 participants and in each sample of patients, the levels of reliability of the 21 items composing of the BDI-II, the eight items composing of the OHHS and the 14 items composing of the OHIP-14-PD varied from good to excellent when they were estimated using the ordinal coefficient alpha (Table 3).

### 3.4. Test of the Normal Distribution Assumption

The transformation of the Napierian logarithm was applied to the values in the 3 biomarkers to correct positive skewness and achieve normality (ln_IL-1β, ln_IL-6, and ln_MMP-8). Except for BDI-II and ln_MMP-8, the distributions were adjusted to normality in the pooled sample of 58 patients (three cases were lost due to invalid biomarker data) by the Kolmogorov–Smirnov test with the Lilliefors correction. The BDI-II presented a slight positive skewness (Z_SK_ = Sk/SE = 3.24), but its distribution was mesokurtic (Z_K3_ = K3/SE = 0.95), which has a minor impact on the estimate of correlations when it is not accomplished the assumption of normal distribution. The distribution of ln_MMP-8 showed symmetry (Z_SK_ = −1.80) and mesokurtosis (Z_K3_ = 0.26), adjusting to a normal distribution by the D’Agostino–Pearson test (*K*^2^ = 3.31, *p* = 0.191) (Table 4).

In the sample of 34 dental patients, 6 of the 7 variables were adjusted to normal by the Shapiro–Wilk test. In the case of BDI-II, the null hypothesis of normality was sustained with a level of significance of 0.01 by this same test. The distribution of scores in BDI-II showed symmetry (Z_SK_ = 0.48), mesokurtosis (Z_K3_ = −1.62), and the null hypothesis of normal distribution was accepted with a significance level of 0.05 by the D’Agostino–Pearson test (*K*^2^ = 2.84, *p* = 0.241).

In the sample of 24 mental health patients, the normality assumption was maintained by the Shapiro–Wilk test with a level of significance at the rate 0.01 in all variables, and at the rate 0.05 in four of them (Table 4).

Regarding multivariate normality, the Mardia’s standardized coefficient of multivariate kurtosis with the seven variables was 2.61 in the pooled sample of 58 patients. Its value was 0.78 in the sample of 34 dental patients and 1.65 in the sample of 24 mental health patients. Therefore, these data reflect an acceptable approximation to multivariate normality in the pooled sample and good in the two patient samples.

### 3.5. Test of the Hypothetical Model in the Pooled Sample of Patients

**Figure 1:**
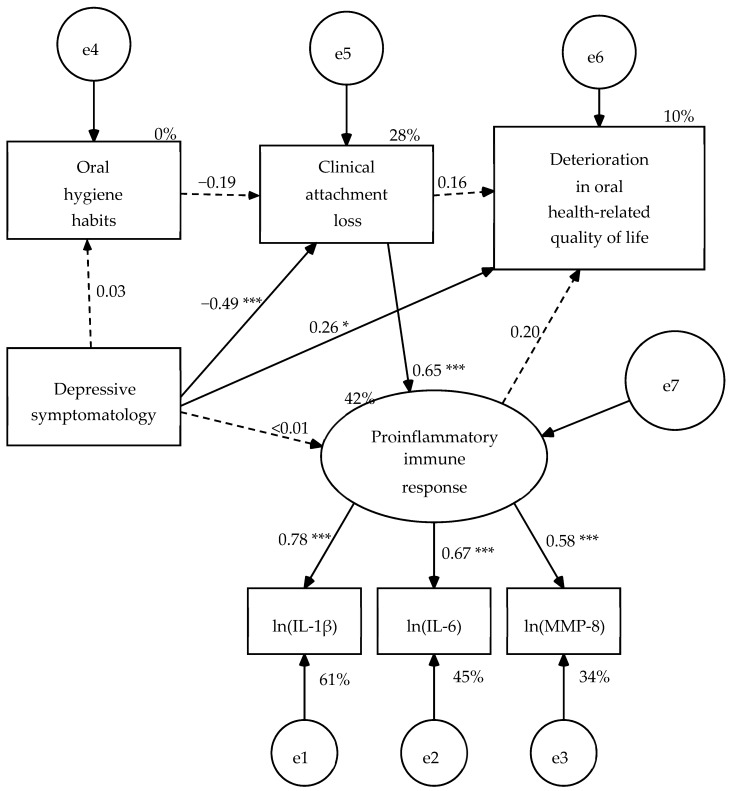
Hypothetical model estimated from the pooled sample of 58 dental and mental health patients. Estimation method: Maximum likelihood. Average variance extracted (AVE) = 0.467 and McDonald’s coefficient ω = 0.721 for the measurement model. Probability value under condition of the null hypothesis was true (H_0_: β = 0) at a two-tailed test: --- *p* > 0.05, * *p* ≤ 0.05, *p* ≤ 0.01, *** *p* ≤ 0.001. ln(IL-1β) = Napierian logarithm of salivary interleukin 1 beta concentration, ln(IL-6) = Napierian logarithm of salivary interleukin 6 concentration, ln(MMP-8) = Napierian logarithm of salivary matrix metalloproteinase-8 concentration, and e = measurement or structural error.

**Figure 2:**
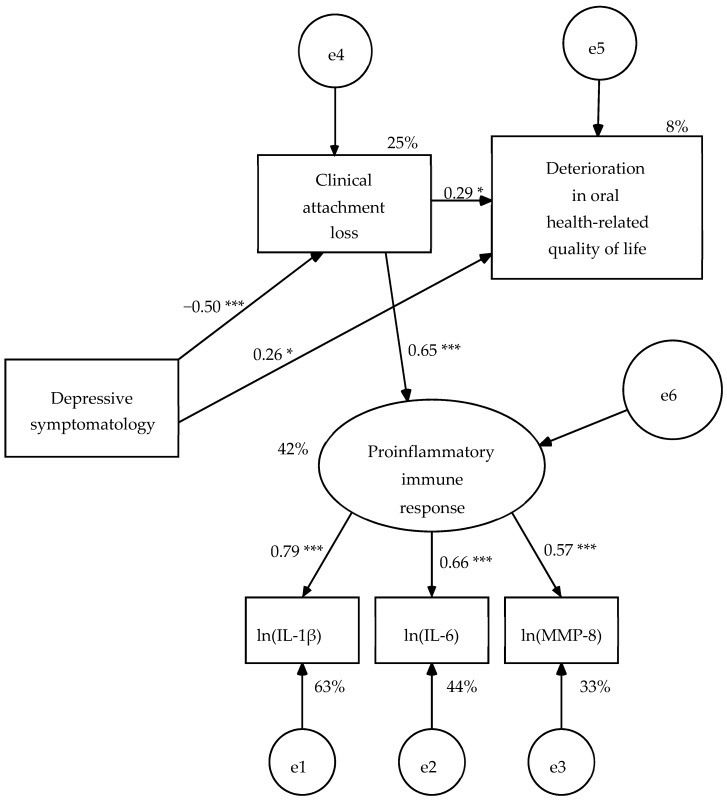
First revision of hypothetical model (R1) estimated from the pooled sample of 58 dental and mental health patients. Estimation method: Maximum likelihood. Average variance extracted (AVE) = 0.465 and McDonald’s coefficient ω = 0.720 for the measurement model. Probability value under condition of the null hypothesis was true (H_0_: β = 0) at a two-tailed test: * *p* ≤ 0.05, *p* ≤ 0.01, *** *p* ≤ 0.001. ln(IL-1β) = Napierian logarithm of salivary interleukin 1 beta concentration, ln(IL-6) = Napierian logarithm of salivary interleukin 6 concentration, ln(MMP-8) = Napierian logarithm of salivary matrix metalloproteinase-8 concentration, and e = measurement or structural error.

A structural model consisting of five variables was specified: one manifest exogenous variable (depressive symptomatology = BDI-II total score), three manifest endogenous variables (oral hygiene habits = OHHS total score, clinical attachment loss, and oral health-related quality of life = OHIP-14-PD total score), and one latent endogenous variable (proinflammatory immune response). The measurement model of this latent variable was made up of three indicators (IL-1β, IL-6, and MMP-8 with their values transformed using Napierian logarithm). In the structural model, depressive symptomatology predicts the 4 endogenous variables, the variable of oral hygiene habits predicts clinical attachment loss, clinical attachment loss predicts proinflammatory immune response and OHRQoL, and finally the proinflammatory immune response predicts OHRQoL (Figure 1). When the parameters were estimated in the pooled sample of 58 patients (three cases were lost due to invalid estimates in the concentration of IL-1β), five structural weights were not significant (Figure 1). Consequently, these paths were eliminated, except for the weight of the clinical attachment loss on the OHRQoL that became significant once the other four structural weights were eliminated (Figure 2).

In this revised model (R1), all structural weights were significant (Figure 2). The AVE of the measurement model was 0.46, its composite reliability (ω) of 0.72 and its measurement weights varied from 0.57 to 0.79, therefore it presented convergent validity. The effect size of the model was large on proinflammatory immunological response (42% of explained variance) and depressive symptomatology (25% of explained variance), and small on OHRQoL (8% of explained variance) (Figure 2). The null hypothesis of goodness of fit was maintained by the likelihood ratio chi-square test (χ^2^[8, *N* = 58] = 6.45, *p* = 0.597) and the Bollen–Stine bootstrap probability value (BS *p* = 0.614). In addition, GFI, IFI, CFI, and RMSEA showed good fit values, and SRMR, AGFI, and NFI reached acceptable fit values (Table 5).

### 3.6. Test of the Invariance of the Model Across the Two Types of Patients Using Multi-Group Analysis

The invariance of the revised model (R1) was tested across dental patients with periodontitis and mental health patients with depressive symptomatology, specifying six nested models in constraints. The solution was admissible in the six nested models in the two samples (34 dental patients and 24 mental health patients). There was a significant difference in two parameters (Table 6). On the one hand, the structural weight of depressive symptomatology on the clinical attachment loss. In the unconstrained model (UC), the weight was negative and significant in dental patients (β = −0.35, *Z* = −2.16, *p* = 0.031), but it was positive and not significant in mental health patients (β = 0.14, *Z* = 0.68, *p* = 0.500). On the other hand, the structural variance of depressive symptomatology was greater in mental health patients (S^2^ = 87.58, 95% CI: 37.15, 138.01) than in dental patients (S^2^ = 17.62, 95% CI: 9.09, 26.14) in the UC model. The model with constraints in all parameters (MR) had two non-significant structural weights: the effect of depressive symptomatology on the clinical attachment loss (β = −0.03, *Z* = −0.24, *p* = 0.812) and on the OHRQoL (*β* = 0.11, *Z* = 0.84, *p* = 0.402).

The null hypothesis of goodness of fit was accepted by the likelihood ratio chi-square test and the Bollen–Stine bootstrap probability value, and χ^2^/df, IFI, CFI, and RMSEA had good fit values in the unconstrained model (UC), as well as in the models with constraints on the measurement weights (MW) and on structural weights (SW) (Table 5). In addition, the goodness of fit was statistically equivalent between these three models nested by the chi-square difference test: χ^2^[2] = 0.15, *p* = 0.929 between UC and MW; χ^2^[6] = 6.97, *p* = 0.324 between UC and SW; χ^2^[4] = 6.82, *p* = 0.146 between MW and SW. However, the fit indices were poor in the models with constraints on structural covariances (SC), structural residuals (SR) and measurement residuals (MR), and the goodness of fit of these three models was significantly lower than that of the previous three models (Table 7).

The R1 model was modified to achieve its partial or incomplete invariance between both populations. First, the non-significant path of the effect of depressive symptomatology on OHRQoL was eliminated. Next, two population singularities were introduced in relation to depressive symptomatology. When reviewing the modification indices, the suggestion of introducing the effect of depressive symptomatology on proinflammatory immunological response in mental health patients was found, which is consistent with the hypotheses. This path was uniquely specified in the sample of mental health patients. The second singularity arose from the fact that the weight of depressive symptomatology on the clinical attachment loss was not significant in mental health patients, but it was significant and with an opposite sign to the expected one in dental patients. Therefore, it was decided to contemplate it in a singular way as a consequence in dental patients and omit it in mental health patients. This proposal as a consequence implies a hypothesis. Dental consultation and diagnosis, probably in the expectation of successful treatment, seem to generate a feeling of euphoria or antidepressant as the patient presents more periodontal injury.

**Figure 3:**
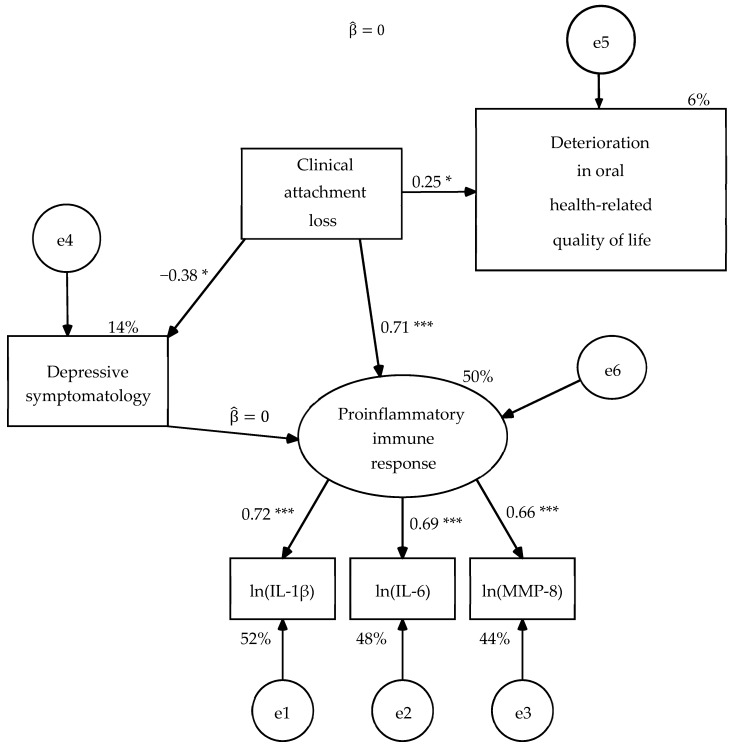
Second revision of hypothetical model (R2) with constraints on measurement residuals (MR) estimated in the sample of 34 dental patients. Estimation method: Maximum likelihood. Average variance extracted (AVE) = 0.477 and McDonald’s coefficient ω = 0.732 for the measurement model. Probability value under condition of the null hypothesis was true (H_0_: β = 0) at a two-tailed test: * *p* ≤ 0.05, *p* ≤ 0.01, *** *p* ≤ 0.001. ln(IL-1β) = Napierian logarithm of salivary interleukin 1 beta concentration, ln(IL-6) = Napierian logarithm of salivary interleukin 6 concentration, ln(MMP-8) = Napierian logarithm of salivary matrix metalloproteinase-8 concentration, and e = measurement or structural error.

**Figure 4:**
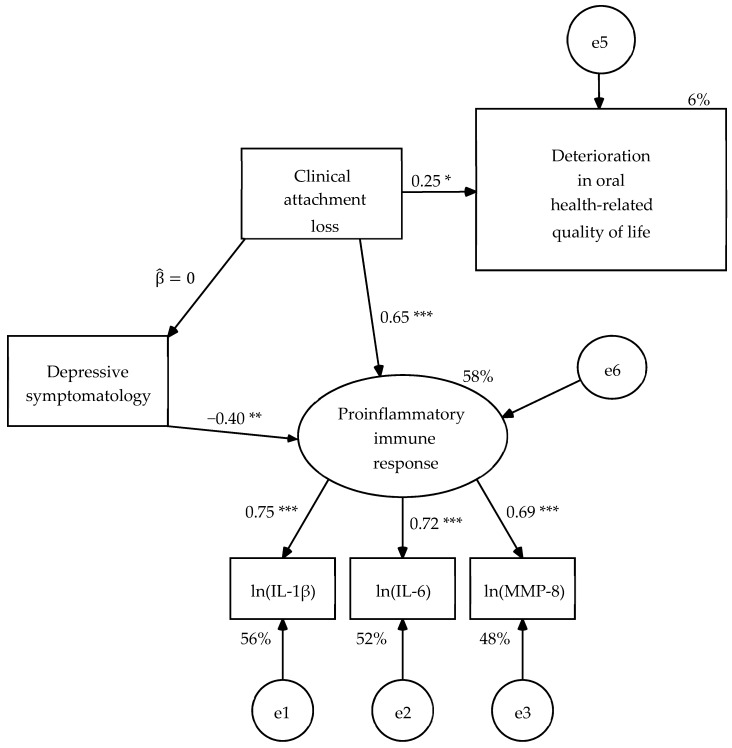
Second revision of hypothetical model (R2) with constraints on measurement residuals (CMR) estimated in the sample of 24 mental health patients. Estimation method: Maximum likelihood. Average variance extracted (AVE) = 0.520 and McDonald’s coefficient ω = 0.765 for the measurement model. Probability value under condition of the null hypothesis was true (H_0_: β = 0) at a two-tailed test: * *p* ≤ 0.05, ** *p* ≤ 0.01, *** *p* ≤ 0.001. ln(IL-1β) = Napierian logarithm of salivary interleukin 1 beta concentration, ln(IL-6) = Napierian logarithm of salivary interleukin 6 concentration, ln(MMP-8) = Napierian logarithm of salivary matrix metalloproteinase-8 concentration, and e = measurement or structural error.

When estimating the parameters of R2 model, the structural weight of the clinical attachment loss on the OHRQoL was significant with significant level of 0.01 in the unconstrained model (UC) and in the model with constraints on the measurement weights (SW) of in both samples, and in the other four nested models with significant level of 0.05 (Figure 3 and Figure 4). The parameters subjected to restriction were statistically equivalent between both types of patients in the nested models (Table 8). The six nested models had good fit to the data (Table 9) and the goodness of fit was equivalent between them by the chi-square difference test (Table 6). Therefore, the R2 model was partially invariant, that is, it was invariant once that a singularity for each population in relation to the depressive symptomatology was specified.

## 4. Discussion

The first objective was to test a model to predict clinical attachment loss and OHRQoL. The model was formulated based on five hypotheses. The first hypothesis was about the convergent validity of proinflammatory immune response as a latent variable, when measured by three biomarkers (IL-1β, IL-6, and MMP-8), that is, it was intended to find out if the data provide evidence that the three indicators assess a same construct. Although Fornell and Larcker in the 1980s proposed that an AVE greater than 0.50 allows us to state that a factor or measurement model has convergent validity, this criterion has been revised; and recently it has been suggested that three criteria must be accomplished to state the convergent validity is acceptably reached: measuring weights greater than or equal to 0.50, coefficient ω or H greater than or equal to 0.70, and AVE not less than 0.25 [18]. In this methodological study, it is shown that when the factor has three indicators, these three conditions are met with an AVE greater than or equal to 0.44 [18]. Measuring weights > 0.50, AVE > 0.44 and ω > 0.70 were conditions satisfied by the latent variable called proinflammatory immune response, and thus the hypothesis was fulfilled. Moreover, we can be affirmed that this measurement model is valid when considering the fact that the levels of IL-1β, IL-6, and MMP-8 were chosen as indicators of this latent variable, since they mediate inflammatory processes and activation of the immune system against periodontal disease [12] and depression [1,3,6].

The second hypothesis to specify the model stated that a higher level of depressive symptomatology would be a predictor of loss of OHRQoL [16], clinical attachment loss [8,9,11], poor oral hygiene habits [13], and increased proinflammatory immune response [1,2]. The hypothesis was fulfilled very partially. It was fulfilled that a higher level of depressive symptomatology predicts worse OHRQoL. Depressive symptomatology also predicted the clinical attachment level, but against the expectation a higher level of depressive symptomatology predicted less clinical attachment loss. On the other hand, depressive symptomatology was independent of oral hygiene habits and proinflammatory immune response. It should be noted that the estimates of these last two structural weights were virtually nil and, thus, it is a clear result and not attributable to a small sample size (n = 58). When combining the first datum consistent with its hypothesis (significant positive effect on OHRQoL), the second discordant with its hypothesis (significant negative effect on clinical attachment loss) and the last two that did not confirm their hypotheses (non-significant weights on oral hygiene habits and proinflammatory immune response), the pooled sample seems to support a psychosomatic model in which there is no correspondence between the patient’s complaint and the evidence that is provided by clinical exams and laboratory tests [40,41]. This same expressed with other words. When both patient samples are analyzed together, the model reflects that people with more depressive symptoms complain of a lower level of OHRQoL as the periodontal damage is less; in addition, their depressive symptoms are independent of the activation of proinflammatory processes and oral hygiene habits.

The third hypothesis to specify the model stated that poor oral hygiene habits are predictors of clinical attachment loss [19]. In the present sample, oral hygiene habits were not a significant predictor with a significance level of 0.05. A very pointed distribution around the maximum value could explain this lack of significance [39]. This would mean that all participants reported very good oral hygiene habits. Nevertheless, this was not the case, since 38% of the participants showed bad habits (OHHS total scores between 0 and 0.999), 57% regular (between 1 and 2.124) and 5% good (between 2.125 and 4). Nor can it be attributed to the lack of reliability of the OHHS, since this was good in the sample of 58 participants (ordinal α = 0.86 and standardized α = 0.80), as in other studies [36]. However, the limited sample size of 58 participants could affect. Altman and Krzywinski [42] point out that when the size of the population effect is small, it may not be significant in small samples [42]. In this regard, in a meta-analysis study, it was found that the size of the effect of oral hygiene habits on clinical attachment loss is small when regular habits dominate: OR = 2.04 (95% CI: 1.65–2.53) [43]. Precisely, if the determination of the statistical significance of the structural weight is performed using a one-tailed test, then the null hypothesis that the parameter is equal to zero would be rejected with a significance level of 0.05 (*Z* = −1.69, *p* = 0.046). Therefore, these data are consistent with the small population effect.

The fourth hypothesis stated that clinical attachment loss predicts an increase in proinflammatory immune response [12] and deterioration of OHRQoL [14]. This hypothesis did fulfill with a large effect size on proinflammatory immunological response and small on OHRQoL. The deterioration of the periodontium due to infection activates the inflammatory and immune response of the organism, and also deteriorates the quality of life of the person [14].

The fifth hypothesis that proposed that the increase in proinflammatory immune response predicts impairment of OHRQoL was not accomplish. As previously noted, the sample size could affect, since the direct effect of the increase in proinflammatory immunological response on OHRQoL is small at the population level and mediated by a complexity of factors [20].

The second objective of the study was to contrast the invariance of the model across the two types of patients: dental patients with periodontitis and mental health patients with depressive symptoms. The model reduced to its significant paths was not strictly invariant and it was decided to define a model that included a singularity in relation to the depressive symptomatology for each population, that is, a partially invariant model. The first singularity introduced in the revision of the model for multi-group analysis was in relation to the effect of depressive symptomatology on clinical attachment level in dental patients. Since depressive symptomatology did not have significant effect in mental health patients, but did have a significant and opposite effect to that expected in dental patients, it was decided to keep this path only in the latter patients, but by reversing its sense. Now a lower depressive symptomatology, which has a minimum level in the sample of dental patients, is a euphoric consequence of the diagnosis of periodontal pathology and the expectation of recovery of oral health [44]. The second singularity introduced in the model was to recover the effect of depressive symptomatology on proinflammatory immune response only in mental health patients, where this path makes more sense [1,3,6].

With the inclusion of the singularity in relation to the depressive symptomatology for each of two patient populations, the six nested models had good fit to the data, the goodness of fit was equivalent between them, and the free estimates of the parameters subject to restriction also they were equivalent between both types of patients (in the nested models in which these parameters were not constrained). This final model indicates that the clinical attachment loss activates proinflammatory immune processes and impairs OHRQoL in both patients, the increase in depressive symptomatology activates proinflammatory immune processes only in mental health patients, and the severity of periodontal tissue damage has a euphoric effect on the situation of periodontal diagnosis and expectation of recovery of oral health only in dental patients. On the other hand, the measurement model for the proinflammatory immune response had convergent validity in all nested models, as in the one-group analysis; moreover the estimates of measurement weights and variance of factor were equivalent between both types of patients.

One limitation of the study is the use of non-probability sampling, hence inferences should be taken with due caution in the populations of dental patients with periodontitis and mental health patients with depressive symptoms, all of them resident in Monterrey and its metropolitan area, middle-aged adults corresponding to a subjective socioeconomic status varying from low to middle-middle. Sample size was limited, which was due to the difficulty of finding mental health patients who met the inclusion and exclusion criteria. This implies that the parameter estimates have to be higher to reach statistical significance; as a consequence, trivial or weak relations at the population level may be non-significant in the sample. However, the end was to find substantial effects with a standardized value of at least 0.20. The design was cross-sectional non-experimental, therefore, no causal inferences can be made and only predictive relationships are discussed. Despite the efforts made in the data analysis to overcome the limitations (verification of assumptions of randomness and multivariate normality, continuous monotonous transformations applied to the data of the three biomarkers to achieve greater proximity to multivariate normality, repetitive sampling procedures with a high number of bootstrap samples to generate errors in the estimates), this research should be considered as a pilot study.

## 5. Conclusions

In the analysis of the pooled sample of dental patients with periodontitis and mental health patients with depressive symptomatology, a psychosomatic complaint model appears. Depressive symptomatology predicts greater complaints or loss of OHRQoL with a small effect size, but less clinical attachment loss with a large effect size, that is, a discordance between the complaint and the clinically observable. On the other hand, depressive symptomatology has no effect on proinflammatory immune response. However, according to expectations, clinical attachment loss predicts the activation of proinflammatory immune processes with a large effect size, as well as a greater complaint of loss of OHRQoL with a small effect size. In addition, the measurement model of this response based on the levels of three proteins (IL-1β, IL-6, and MMP-8) shows convergent validity. The effect of bad oral hygiene habits on the clinical attachment loss is not significant in a two-tailed test and, thus, this path was eliminated, although this effect of small size would be significant in a one-tailed test. The model reduced to its significant paths shows good fit to the data in the one-group analysis, but is not invariant between both types of patients.

In the multi-group analysis, the effect of depressive symptomatology on OHRQoL is not significant in both a one-tailed test and a two-tailed test. Although the effect of the clinical attachment loss on the OHRQoL is not significant in two nested models, it is significant in the four models with more constraints, hence, the elimination of this path is not justified. When specifying a singularity in relation to depressive symptomatology for each population, a model with a good fit to the data and a goodness of fit equivalent between its six specifications nested in constraints is achieved; moreover, parameters subject to constrain in each nested model were statistically equivalent between both groups of patients. Due to this singularity for each population, it should be named as a strong but partial invariance. A singularity refers to the effect of depressive symptomatology on the clinical attachment loss disappears in patients with depressive symptomatology and its sense is reversed in dental patients, that is, it is contemplated as a euphoric consequence of diagnosis and expectations on treatment in dental patients with periodontitis. The other singularity is with respect to the effect of depressive symptomatology on proinflammatory immune response, eliminated due to lack of significance in the one-group analysis, is recovered for the sample of patients with depressive symptomatology in the multi-group analyses. The common paths between both types of patients in the final model are the large-size effect of clinical attachment loss on the increase in proinflammatory immune response and the small-size effect of clinical attachment loss on OHRQoL. As in the one-group analysis (when pooling both samples), the measurement model of this latent variable has convergent validity and its measurement weights and variance are equivalent between both types of patients.

## Figures and Tables

**Table 1:** Frequency distributions of sociodemographic variables and comparisons between the two samples of patients.

Variable		DP	MHP	Pooled	Test	
		*n* (%)	*n* (%)	*n* (%)	T	*p*
**Sample**		**35 (38.9%)**	**26 (28.9%)**	**61 (100%)**	**B**	**0.306**
Sex	Women	16 (45.7%)	15 (57.7%)	31 (50.8%)	χ^2^	0.505
	Men	19 (54.3%)	11 (42.3%)	30 (49.2%)		
Age (years old)	35–39	6 (17.1%)	8 (30.8%)	14 (23%)	t	0.159
	40–49	14 (40%)	12 (46.2%)	26 (42.6%)		
	50–59	12 (34.3%)	5 (19.2%)	17 (27.9%)		
	60–65	3 (8.6%)	1 (3.8%)	4 (6.6%)		
Educational level	Primary	5 (14.3%)	3 (11.5%)	8 (13.1%)	Z_U_	0.958
	Secondary	9 (25.7%)	10 (38.5%)	19 (31.1%)		
	High school	6 (17.1%)	1 (3.8%)	7 (11.5%)		
	Vocational	7 (20%)	5 (19.2%)	12 (19.7%)		
	Bachelor	7 (20%)	5 (19.2%)	12 (19.7%)		
	Post-graduate	1 (2.9%)	2 (7.7%)	3 (4.9%)		
Subjective socioeconomic status	Low	2 (5.7%)	2 (7.7%)	4 (6.6%)	Z_U_	0.191
	Middle-low	12 (34.3%)	13 (50%)	25 (41%)		
	Middle-middle	21 (60%)	11 (42.3%)	32 (52.4%)		
Civil status	Married	25 (71.4%)	13 (50%)	38 (62.3%)	χ^2^	0.177
	Single	2 (5.7%)	6 (23.1%)	8 (13.1%)		
	Divorced or separated	3 (8.6%)	5(19.2%)	8 (13.1%)		
	Cohabitating	3 (8.6%)	1 (3.8%)	4 (6.6%)		
	Willow	2 (5.7%)	1 (3.8%)	3 (4.9%)		
Occupation	White collar	15 ^a^ (42.9%)	13 ^a^ (50%)	28 (45.9%)	χ^2^	0.030
	Housewife	11 ^a^ (31.4%)	12 ^a^ (46.2%)	23 (37.7%)		
	Blue collar	8 ^a^ (22.9%)	0 ^b^ (0%)	8 (13.1%)		
	Other *	1 (5.7%)	1 (3.8%)	2 (3.3%)		

Note. *n* = absolute frequency and % = percentage. Samples: DP = dental patients with periodontitis, MHP = mental health patients with depressive symptomatology. Test: T = test statistic (B = binomial test, χ^2^ = Pearson’s chi-square test, t = Student’s *t*-test, Z_U_ = Mann–Whitney U-test), *p* = probability value under the condition that the null hypothesis is true at a two-tailed test; ^a,b^ = each table superscript letter denotes a subset of categories whose row values do not differ significantly from each other at the 0.05 level of significance applying Bonferroni correction for multiple comparisons. * The category of “other occupation” was excluded in the calculation of the test.

**Table 2:** Runs test for the randomness of the data sequence.

Variables	DP (*n* = 35)				MHP (*n* = 26)				Pooled Sample (*n* = 61)				
	*n*_0_	*n*_1_	*R*	*p*	*n*_0_	*n*_1_	*R*	*p*	*n*_0_	*n*_1_	*R*	*Z*	*p*
Sex ^c^	16	19	17	0.730	15	11	12	0.544	31	30	29	−0.644	0.520
Civil status ^a^	2	33	5	1	6	20	9	0.562	8	53	13	−1.100	0.271
Occupation	18	17	15	0.303	12	14	10	0.160	31	28	25	−1.428	0.153
Schooling	14	21	19	0.724	13	13	13	0.836	27	34	34	0.759	0.448
SSES ^b^	14	21	19	0.724	15	11	13	0.837	29	32	33	0.407	0.684
Age	19	16	21	0.390	12	14	15	0.692	30	31	29	−0.644	0.520
BDI-II ^c^	15	20	14	0.164	14	12	16	0.428	38	23	26	−1.006	0.314
OHHS ^c^	16	19	15	0.300	14	12	18	0.065	31	30	27	−1.160	0.246
OHIP-14-PD ^c^	21	14	13	0.107	13	13	16	0.554	32	29	34	0.666	0.505
CAL ^c^	19	16	14	0.166	12	14	12	0.551	28	33	22	−2.417	0.016
IL-1β ^c^	22	12	14	0.341	20	4	6	0.163	43	15	16	−2.515	0.012
IL-6 ^c^	22	13	15	0.462	16	8	11	0.829	39	20	24	−1.010	0.312
MMP-8 ^c^	22	13	21	0.204	15	9	8	0.060	36	23	30	0.258	0.797

Note. Cut-off point: ^a^ = mode, ^b^ = medium, and ^c^ = arithmetic mean. Statistics: *n*_0_ = number of cases < arithmetic mean or median, *n*_1_ = number of cases ≥ arithmetic mean or median, *R* = number of runs, and *p* = exact two-tailed probability value. Samples: DP = dental patients with periodontitis, MHP = mental health patients with depressive symptomatology, and Pooled sample = union of two patient samples. Variables: SSES = subjective socioeconomic status, BDI-II = total score in the Beck Depression Inventory-II, OHHS= total score in the Oral Hygiene Habits Scale, OHIP-14-PD = total score in the Oral Health Impact Profile applied to Periodontal Disease, CAL = clinical attachment loss, IL-1β = salivary interleukin 1 beta concentration, IL-6 = salivary interleukin 6 concentration, and MMP-8 = salivary matrix metalloproteinase-8 concentration.

**Table 3:** Internal consistency reliability through the ordinal coefficient alpha.

Scales and Cytokine Concentration in Salivary Samples	Statistic	Sample		
		DP (*n* = 35)	HMP (*n* = 26)	Pooled (*n* = 61)
BDI-II (21 items)	ordinal α	0.874	0.856	0.957
OHHS (8 items)	ordinal α	0.802	0.913	0.861
OHIP-14-PD (14 items)	ordinal α	0.912	0.936	0.923
IL-1β	r [95% CI]			0.998 [0.994, 1]
IL-6	r [95% CI]			0.999 [0.997, 1]
MMP-8	r [95% CI]			0.972 [0.955, 1]

Note. Sample: DP = dental patients with periodontitis, MHP = mental health patients with depressive symptomatology, and Pooled = union of the two patient samples. Scales: BDI-II = total score in Beck Depression Inventory-II, OHHS = total score in Oral Hygiene Habits Scale, and OHIP-14-PD = total score in the Oral Health Impact Profile applied to Periodontal Disease. Cytokines: IL-1β = salivary interleukin 1 beta concentration, IL-6 = salivary interleukin 6 concentration, and MMP-8 = salivary matrix metalloproteinase-8 concentration. Statistic: *n* = sample size, ordinal α = standardized coefficient alpha calculated from polychoric correlation matrix, r = Pearson’s product-moment correlation coefficient between eight levels of serial dilutions of the antibody concentration (0, 3.125, 6.25, 12.5, 25, 50, 100, and 200 pg/mL for IL-1β; 0.156, 3.125, 6.25, 12.5, 25, 50, and 100 pg/mL for IL-6; and 0, 78, 156, 313, 625, 1250, 2500, and 5000 pg/mL for MMP-8) and the average absorbance (negative of the logarithm with base 10 of quotient between incident and transmitted light intensities). Concentration and absorbance values are only available for each microplate; since the microplate of each cytokine test has 96 wells, both patient samples were joined and, therefore, correlations could only be calculated in the pooled sample. CI = confidence interval calculated through percentile bootstrapping method with the simulation of 1000 random samples.

**Table 4:** Tests for univariate normality.

Variables	Pooled Sample (*n* = 58)				Dental Patients (*n* = 34)				Mental Health Patients (*n* = 24)			
	*D*	*p*	*K* ^2^	*p*	*W*	*p*	*K* ^2^	*p*	*W*	*p*	*K* ^2^	*p*
OHHS	0.11	0.089	25.36	<0.001	0.96	0.238	0.67	0.715	0.90	0.021	17.48	<0.001
BDI-II	0.14	0.006	11.36	0.003	0.92	0.013	2.84	0.241	0.92	0.045	4.29	0.117
OHIP	0.11	0.088	3.44	0.179	0.95	0.101	11.97	0.003	0.94	0.168	1.85	0.397
CAL	0.07	0.200	25.36	<0.001	0.96	0.170	0.67	0.715	0.97	0.573	17.48	<0.001
IL-1β	0.26	<0.001	345.34	<0.001	0.72	<0.001	345.34	<0.001	0.54	<0.001	345.34	<0.001
IL-6	0.19	<0.001	170.87	<0.001	0.78	<0.001	170.87	<0.001	0.68	<0.001	170.87	<0.001
MMP-8	0.16	0.001	27.30	<0.001	0.81	<0.001	27.30	<0.001	0.79	<0.001	27.30	<0.001
ln(IL-1β)	0.11	0.082	1.06	0.590	0.95	0.165	4.85	0.088	0.89	0.012	8.32	0.016
ln(IL-6)	0.09	0.200	0.63	0.730	0.98	0.637	0.02	0.990	0.96	0.393	3.57	0.168
ln(MMP)	0.15	0.002	3.31	0.191	0.95	0.085	1.60	0.449	0.96	0.378	2.76	0.252

Note. Variables: BDI-II = total score in the Beck Depression Inventory-II, OHHS = total score in the Oral Hygiene Habits Scale, OHIP-14-PD = total score in the Oral Health Impact Profile applied to Periodontal Disease, IL-1β = salivary interleukin 1 beta concentration, CAL = clinical attachment loss = mean of sites with insertion loss (≥1 mm) in each participant, IL-6 = salivary interleukin 6 concentration, MMP-8 = salivary matrix metalloproteinase-8 concentration, ln(IL-1β) = Napierian logarithm of IL-1β, ln(IL-6) = Napierian logarithm of IL-6, and ln(MMP) = Napierian logarithm of MMP-8. Statistics: *D* = Kolmogorov–Smirnov test statistic with the Lilliefors correction when calculating the probability under null hypothesis of normal distribution, *W* = Shapiro–Wilk test statistic, *K*^2^ = D’Agostino–Pearson test statistic, and *p* = probability value under null hypothesis that scores follow a normal distribution.

**Table 5:** Fit indices in the one-group analysis and the multi-group analysis across dental and mental health patients for the first revision of the hypothetical model (R1).

Fit Indices		One-Group	Multi-Group Analysis					
			UC	MW	SW	SC	SR	MR
χ^2^		6.451	18.106	18.254	25.073	42.347	44.398	51.933
df		8	16	18	22	23	26	29
*p*		0.597	0.318	0.439	0.294	0.008	0.014	0.006
χ^2^/df		0.806	1.132	1.014	1.140	1.841	1.708	1.791
BS *p*		0.614	0.331	0.455	0.310	0.010	0.026	0.011
GFI		0.962	0.915	0.914	0.880	0.799	0.801	0.781
IFI		1	0.971	0.996	0.954	0.708	0.709	0.619
CFI		1	0.964	0.996	0.948	0.673	0.689	0.612
RMSEA (90% CI)	Point value	0	0.048	0.016	0.050	0.123	0.112	0.119
	LB	0	0	0	0	0.061	0.051	0.064
	UB	0.134	0.137	0.120	0.126	0.180	0.168	0.170
	*p*-close	0.689	0.462	0.593	0.461	0.030	0.048	0.025
SRMR		0.060	0.073	0.072	0.132	0.126	0.130	0.122

Note. Nested models in constrains for multi-group analysis: UC = Unconstrained model, MW = with constraints on the measurement weights, SW = with constraints on the structural weights, SC = with constraints on the structural covariances, SR = with constrains on the structural residuals, and MR = with constraints on measurement residuals. Fit indices: χ^2^ = likelihood ratio chi-square statistic, df = degrees of freedom for the likelihood ratio chi-square test, *p* = probability value under null hypothesis of goodness of fit for the likelihood ratio chi-square statistic, χ^2^/df = relative chi-square, BS *p* = Bollen–Stine bootstrap probability value (with the simulation of 1000 random samples), GFI = Jöreskog-Sörbom goodness-of-fit index, IFI = incremental fit index through Bollen’s coefficient delta-2, CFI = Bentler’s comparative fit index, RMSEA (90% CI) = point estimation and interval estimate with 90% confidence level for Steiger–Lind root mean square error of approximation, LB = lower boundary and UB = upper boundary of a two-sided 90% confidence interval for population RMSEA, *p*-close = probability value under null hypothesis of close fit: H_0_ = RMSEA ≤ 0.05, and SRMR = standardized root mean square residuals.

**Table 6:** Significance of parameters in the model with constraints on measurement residuals and difference in parameters with constraints between both patients in each nested model for the first revision of hypothetical model (R1).

Parameter	PE in MR	UC		MW		SW		SV		SR	
		*Z*	*p*	*Z*	*p*	*Z*	*p*	*Z*	*p*	*Z*	*p*
β_PIR → IL1_	0.74	Id									
β_PIR → IL6_	0.69 ***	−0.40									
β_PIR → MMP8_	0.67 ***	−0.24	0.813								
β_BDI → CAL_	−0.03 ^ns^	2.17	0.030	2.17	0.030						
β_BDI → OHIP_	0.11 ^ns^	−0.53	0.595	−0.53	0.595						
Β_CAL → AIP_	0.67 *	−1.05	0.296	−1.43	0.154						
Β_CAL → OHIP_	0.27 *	−0.19	0.849	−0.19	0.849						
σ^2^_BDI_	46.57 ***	2.68	0.007	2.68	0.007	2.68	0.007				
σ^2^_ε_IL-1β_	0.65 ***	−1.75	0.081	−1.71	0.087	−1.81	0.070	−1.81	0.070		
σ^2^_ε_IL-6_	0.26 ***	−0.61	0.543	−0.80	0.427	−0.67	0.504	−0.67	0.504		
σ^2^_ε_MMP-8_	0.47 ***	−1.18	0.238	−1.31	0.189	−1.22	0.223	−1.22	0.223		
σ^2^_ε_CAL_	0.13 ***	1.07	0.284	1.07	0.284	0.98	0.325	0.98	0.325	−1.36	0.174
σ^2^_ε_OHIP_	118.04 ***	0.60	0.551	0.60	0.551	0.59	0.558	0.59	0.558	−1.97	0.049
σ^2^_ε_PIR_	0.43 *	−0.43	0.671	−0.74	0.457	−0.86	0.391	−0.86	0.391	−0.84	0.399

Note. Parameter: β = measurement or structural weight, σ^2^ = structural variance, and σ^2^ε = error variance. Id = Parameter identified or set to 1 in both samples = β_PIR_ → IL1β. Statistic: PE in MR = point estimation in the nested model with constraints on measurement residuals (statistical significance of the parameter at a two-tailed test: ns = non-significance *p* > 0.05, * *p* ≤ 0.05, *p* ≤ 0.01, *** *p* ≤ 0.001), *Z* = Z-test statistic, *p* = probability value at a two-tailed test. Nested models: UC = unconstrained model, MW = model with constraints on the measurement weights, SW = model with constraints on the structural weights, SC = model with constrains on the structural covariances, and SR = model with constrains on the structural residuals. Variables: PIR = proinflammatory immune response, BDI = total score in the Beck Depression Inventory-II, CAL = clinical attachment loss = mean of sites with insertion loss (≥1 mm) in each participant, IL-1β = Napierian logarithm of salivary interleukin 1 beta concentration, IL-6 = Napierian logarithm of salivary interleukin 6 beta concentration, and MMP-8 = Napierian logarithm of salivary matrix metalloproteinase-8 concentration.

**Table 7:** Goodness-of-fit comparison between nested models.

Difference between Models	First Revision of Hypothetical Model (R1)			Second Revision of Hypothetical Model (R2)		
	Δχ^2^	Δdf	*p*	Δχ^2^	Δdf	*p*
UC−MW	0.148	2	0.929	0.007	2	0.997
UC−SW	6.967	6	0.324	1.472	4	0.832
UC−SC	24.241	7	0.001	2.219	5	0.818
UC−SR	26.292	10	0.003	5.095	7	0.648
UC−MR	33.827	13	0.001	13.294	10	0.208
MW−SW	6.819	4	0.146	1.465	2	0.481
MW−SC	24.093	5	<0.001	2.212	3	0.530
MW−SR	26.144	8	0.001	5.088	5	0.405
MW−MR	33.679	11	<0.001	13.287	8	0.102
SW−SC	17.274	1	<0.001	0.746	1	0.388
SW−SR	19.325	4	0.001	3.622	3	0.305
SW−MR	26.86	7	<0.001	11.821	6	0.066
SC−SR	2.051	3	0.562	2.876	2	0.237
SC−MR	9.585	6	0.143	11.075	5	0.050
SR−MR	7.535	3	0.057	8.199	3	0.042

Note. Nested models in constraints: UC = Unconstrained model, MW = model with constraints on the measurement weights, SW = model with constraints on the structural weights, SC = model with constrains on the structural covariances, SR = model with constrains on the structural residuals, and MR = model with constraints on measurement residuals. Statistic: Δχ^2^ = chi-square difference test statistic, Δdf = difference between degrees of freedom of the two models being compared, and *p* = probability value of chi-square difference statistic under null hypothesis of equality of goodness of fit between the two models being compared.

**Table 8:** Difference in parameters with constraints between both patient groups in each nested model for the second revision of hypothetical model (R2).

Parameter	UC		MW		SW		SC		MR	
	*Z*	*p*	*Z*	*p*	*Z*	*p*	*Z*	*p*	*Z*	*p*
β_PIR → IL1β_	Id									
β_PIR → IL6_	−0.034	0.973								
β_PIR → MMP8_	0.056	0.955								
Β_CAL → PIR_	−1.019	0.308	−1.224	0.221						
Β_CAL → OHIP_	0.074	0.941	0.074	0.941						
σ^2^_CAL_	0.817	0.414	0.817	0.414	0.817	0.414				
σ^2^_ε_IL−1β_	−1.463	0.143	−1.547	0.122	−1.658	0.097	−1.657	0.098		
σ^2^_ε_IL−6_	−0.786	0.432	−0.842	0.400	−0.722	0.470	−0.722	0.470		
σ^2^_ε_MMP−8_	−1.278	0.201	−1.306	0.192	−1.214	0.225	−1.214	0.225		
σ^2^_ε_CAL_	−1.182	0.237	−1.391	0.164	−1.463	0.143	−1.459	0.145	−0.843	0.399
σ^2^_ε_OHIP_	0.563	0.573	0.563	0.573	0.563	0.573	0.563	0.573	−0.933	0.351

Note. Parameter: β = measurement or structural weight, σ2 = structural variance, σ2ε = error variance. Id = Parameter identified or set to 1 in both samples = βPIR → IL1β. Free or unrestricted parameters between samples: βCAL → BDI and σ2ε_BDI in dental patients, as well as βBDI → PIR and σ2BDI in mental health patients. Statistic: *Z* = Z-test statistic, *p* = probability value at a two-tailed test. Nested models: UC = unconstrained model, MW = model with constraints on the measurement weights, SW = model with constraints on the structural weights, SC = model with constrains on the structural covariances, and SR = model with constrains on the structural residuals. Variables: PIR = proinflammatory immune response, BDI = total score in the Beck Depression Inventory-II, CAL = clinical attachment loss = mean of sites with insertion loss (≥1 mm) in each participant, IL-1β = Napierian logarithm of salivary interleukin 1 beta concentration, IL-6 = Napierian logarithm of salivary interleukin 6 beta concentration, and MMP8 = Napierian logarithm of salivary matrix metalloproteinase-8 concentration.

**Table 9:** Fit indices in the multi-group analysis across dental and mental health patients for the second revision of the hypothetical model (R2).

Fit Indices		UC	MW	SW	SC	SR	MR
χ^2^		12.889	12.896	14.361	15.107	17.983	26.182
df		18	20	22	23	25	28
*p*		0.798	0.882	0.888	0.891	0.843	0.563
χ^2^/df		0.716	0.645	0.653	0.657	0.719	0.935
BS *p*		0.902	0.945	0.942	0.935	0.918	0.800
GFI		0.932	0.932	0.927	0.924	0.909	0.878
IFI		1	1	1	1	1	1
CFI		1	1	1	1	1	1
RMSEA (90% CI)	Point value	0	0	0	0	0	0
	LB	0	0	0	0	0	0
	UB	0.078	0.057	0.053	0.052	0.063	0.095
	*p*-close	0.883	0.939	0.945	0.947	0.923	0.737
SRMR		0.079	0.079	0.097	0.090	0.092	0.077

Note. Nested models in constrains for multi-group analysis: UC = Unconstrained model, MW = model with constraints on the measurement weights, SW = model with constraints on the structural weights, SC = model with constrains on the structural covariances, SR = model with constrains on the structural residuals, and MR = model with constraints on measurement residuals. Fit indices: χ^2^ = likelihood-ratio chi-square statistic, df = degrees of freedom for the likelihood-ratio chi-square test, *p* = probability value under null hypothesis of goodness of fit at the likelihood-ratio chi-square test, χ^2^/df = relative chi-square, BS *p* = Bollen–Stine bootstrap probability value (with the simulation of 1000 random samples), GFI = Jöreskog-Sörbom goodness-of-fit index, IFI = incremental fit index through Bollen’s coefficient delta-2, CFI = Bentler’s comparative fit index, RMSEA (90% CI) = point estimation and interval estimate with 90% confidence level for Steiger-Lind root mean square error of approximation, LB = lower boundary and UB = upper boundary of a two-sided 90% confidence interval for population RMSEA, *p*-close = probability value under null hypothesis of close fit: H_0_ = RMSEA ≤ 0.05, and SRMR = standardized root mean square residuals.
